# Identifying and overcoming barriers and facilitators to blood donation in young adults using the theoretical domains frameworks

**DOI:** 10.1177/13591053251346387

**Published:** 2025-06-30

**Authors:** Velina Hristova, Freya Mills, Ivo Vlaev

**Affiliations:** 1 Bulgarian Academy of Sciences, Bulgaria; 2 Sofia University, Bulgaria; 3 University College London, UK; 4 University of Sussex, UK; 5 University of Warwick, UK

**Keywords:** behavior change wheel, behavioral science, blood donation, digital engagement, intervention development, theoretical domains framework, young donors

## Abstract

This study applied the Theoretical Domains Framework (TDF) to identify barriers and facilitators to blood donation among young adults in the UK. A total of 195 individuals (aged 18–29) completed an online survey covering 14 TDF domains, with non-donors offered the chance to register as donors. Binary logistic regression analysis revealed that Knowledge, Beliefs about capabilities and Emotion were the most significant predictors of current donation status. Although nearly half of the non-donors expressed interest in registering as donors, only about a quarter completed the registration when provided with a link. The TDF proved to be an effective framework for understanding the psychological and behavioral factors influencing donation decisions. Based on these findings, targeted intervention strategies were suggested using the Behavior Change Wheel (BCW). These approaches emphasize digital engagement, aligning with the online behaviors and social influences that shape young adults’ decision-making. Further research is needed to implement and evaluate these interventions, comparing their effectiveness against current NHS Blood and Transplant (NHSBT) campaigns.

Blood is a resource available to all, yet less than 4% of the eligible UK population donate (Ferguson et al., 2019). In England, 140,000 new blood donors are needed every year (National Health Service Blood and Transplant Unit (NHSBT), 2023), yet, in 2019 the number of new donors was 131,825—the lowest figure in more than 5 years.

This issue is not unique to the UK. Globally, 118.5 million blood donations are collected each year, yet 40% come from high-income countries, which account for just 16% of the world’s population (World Health Organisation (WHO), 2020). In the EU, blood donor rates vary significantly, with the average rate among 22 member states at 2.41%—ranging from as low as 1.43% in Ireland to 6.46% in Cyprus (Vuletić et al., 2023). Many countries struggle to reach even a 3% donor rate, highlighting a widespread challenge in ensuring an adequate blood supply.

Young blood donors represent an ideal target population for increasing donation rates due to several key advantages. First, they have the potential for longer donor careers, meaning that engaging them early can lead to a sustained and consistent blood supply over time. Encouraging young individuals to establish a habit of donating can significantly contribute to long-term donor retention and overall blood availability. Additionally, younger donors are generally healthier and less likely to have medical conditions that would prevent them from donating, such as chronic illnesses that become more prevalent with age (NHSBT, 2020). Given that the number of young people donating blood is alarmingly low, with 81% of 18–24-year-olds in the UK never having donated (NHSBT, 2018), targeted interventions to engage this demographic are essential.

## Blood donation intention

A variety of factors influence blood donation intentions, with psychological, social, and situational variables shaping individuals’ willingness to donate. The Theory of Planned Behavior (TPB) is one of the most widely applied frameworks for understanding blood donation behavior (Bednall et al., 2013; White et al., 2023). Developed as an extension of Fishbein and Ajzen’s (1975) Theory of Reasoned Action, the TPB provides a well-established theoretical model of behavior change (Ajzen, 1991). According to the theory, behavior is primarily driven by behavioral intention, itself determined by attitude (one’s evaluation of donating), subjective norms (perceived social pressure to donate), and perceived behavioral control (the individual’s confidence in their ability to donate). While TPB has been effective in predicting donor intention (Holdershaw et al., 2011), researchers have found that extending the model to include additional constructs enhances its predictive power.

An early meta-analysis by Bednall et al. (2013) investigated the effectiveness of TPB and its extensions in predicting blood donation behavior and found that the strongest correlates of donation intention were perceived behavioral control, attitude, self-efficacy, role identity, and anticipated regret. Self-efficacy, or the belief in one’s ability to successfully donate, emerged as an important determinant, suggesting that individuals who feel more confident in their capacity to donate are more likely to do so. Anticipated regret, or the emotional discomfort associated with failing to donate, also played a key role in shaping intentions. Another significant predictor was role identity, which refers to how strongly an individual perceives being a blood donor as part of their self-concept. Together, these results highlight that, beyond traditional TPB constructs, both personal agency and emotions related to donation play a critical role in influencing behavior.

Building on these insights, Mugion et al. (2021) proposed an even more comprehensive approach by incorporating additional situational and service-related factors into the TPB framework. Their study highlighted the importance of service quality—including the kindness and competence of medical staff, waiting times, cleanliness of transfusion centers, and accessibility of donation information—as a major influence on donor intentions. Additionally, they emphasized the central role of information and communication in blood donation promotion. They also investigated inhibitors such as fear of needles, pain, fainting, and the sight of blood, but found that these factors were not significant direct predictors of donation intention. Instead, among existing donors, an awareness of the importance of blood donation outweighed these concerns.

Further research has examined the role of anxiety-related inhibitors in blood donation, consistently demonstrating that anxiety does not directly influence donation intention but operates through mediating psychological mechanisms. France and France (2018) found that potential donors greatly overestimated the risk of fainting and prefaint reactions (e.g. dizziness, lightheadedness), with perceived fainting risk over 20 times higher than the actual risk. Those with greater fear of blood draws reported even higher perceived risks, highlighting anxiety as a key deterrent to donation. Another more recent study indicated that the negative relationship between blood donation anxiety and donation intention was mediated by moral disengagement (the process of justifying inaction through moral reasoning; Chen et al., 2022). Additionally, mindfulness was found to buffer both the direct and indirect effects of anxiety, reducing moral disengagement and improving donation intention. A further study using an extended TPB model among young Chinese university students (Liu and Han, 2023) confirmed that anxiety influences donation intention only through indirect pathways. Their findings revealed that attitude and self-efficacy were significant positive predictors of blood donation intention. This means that while anxiety was present, it only affected donation intention indirectly by shaping individuals’ attitudes toward donation and their perceived ability to donate. Taken together, these studies consistently demonstrate that anxiety is a significant psychological barrier to blood donation, particularly for non-donors, but it does not exert a direct influence on donation intention. Instead, its effects are mediated through mechanisms such as risk perception, moral disengagement, attitude, and self-efficacy.

More recently, White et al. (2023) conducted a meta-analysis examining the broader determinants of charitable decisions, including blood donation. Their findings identified perceived behavioral control, moral norms (personal beliefs about the ethical obligation to donate), attitude, and subjective norms (perceived expectations from others regarding donation behavior) as key predictors of prosocial behavior. However, they also found that subjective norm-intention (the extent to which perceived social expectations influence a person’s decision to donate) and moral norm-intention (the extent to which personal moral beliefs influence the decision to donate) relationships were weaker for blood donation compared to other charitable acts. The authors attributed this to the solitary nature of blood donation, which lacks the immediate social reinforcement or visibility found in other prosocial behaviors such as volunteering or fundraising. Unlike these activities, which often involve public acknowledgment and shared participation, blood donation typically occurs in private, reducing the influence of social pressure as a motivator.

Beyond psychological and emotional factors, contextual barriers also influence donor behavior. Piersma et al. (2021) investigated the impact of donation center closures on donor retention and found that individuals whose nearest donation center closed were 53% more likely to lapse compared to those whose center remained open. Furthermore, the likelihood of donor lapse increased with each extra kilometer of distance to the new nearest donation center. These findings underscore the importance of environmental context in donor retention, suggesting that reducing physical barriers to donation, such as increasing mobile donation units or ensuring a more even distribution of centers, could help maintain donor engagement.

Together, these studies demonstrate that while TPB remains a valuable foundation for understanding blood donation behavior, its predictive power is significantly enhanced by incorporating additional psychological, emotional, and contextual factors.

## Blood donation interventions

Understanding blood donation behavior extends beyond merely examining donors’ intentions, as research consistently demonstrates that intentions do not always translate into actual behavior, highlighting a notable intention-behavior gap (White et al., 2023). This gap suggests that while individuals may express a willingness to donate, various psychological, contextual, and environmental factors may prevent them from following through. Therefore, intervention studies are crucial in identifying strategies that effectively translate intentions into actual donations.

Intervention studies provide valuable insights into real-world blood donation behavior. An early systematic review revealed that interventions targeting psychosocial cognitions, particularly those emphasizing altruistic motivation, were among the most successful strategies for increasing blood donation rates (Godin et al., 2012). Beyond psychological interventions, reminder-based and motivational strategies have also proven effective in enhancing actual donor behavior. Studies have demonstrated that telephone and email reminders significantly increase blood donation rates by keeping the need for blood donations salient (Germain and Godin, 2015). Similarly, educational interventions tailored to potential donors’ concerns have been shown to boost donor commitment and willingness to donate (Sutton, 2017). Cognition-based and motivational interventions—which target donor attitudes, perceived behavioral control, and intrinsic motivation—also play a critical role in encouraging repeat donations (Makin et al., 2019). Additionally, fostering social spillovers, where existing donors influence their peers to donate, has been identified as another effective method in expanding the donor pool (Bruhin et al., 2020).

Finally, recent research has also explored the role of technology-driven interventions in blood donor management, particularly in regions where blood supply shortages are persistent. Müller and Reuter-Oppermann (2024) examined the application of Blood Donation Behavior Change Support Systems (BDBCSS) via a blood donation app and a chatbot, finding that these systems can be beneficially applied in African countries, where meeting the demand for blood products is already a major challenge. Their research underscores the potential for digital interventions to play a transformative role in improving blood donation infrastructure, particularly in resource-limited settings.

## Theoretical domains framework

This study aims to build upon previous research by exploring the barriers and facilitators influencing blood donation behavior among young donors, with the goal of integrating a comprehensive theoretical framework into intervention development. Specifically, it will employ the Theoretical Domains Framework (TDF; Cane et al., 2012), a framework embedded within the Behavior Change Wheel (BCW; Michie et al., 2014), to provide a broader and more integrative approach to behavior change than single-theory models such as TPB.

Unlike TPB, which primarily focuses on intention, attitude, subjective norms, and perceived behavioral control, the TDF consolidates 128 constructs from 33 behavior change theories into 14 domains that encompass cognitive, emotional, social, and environmental influences on behavior. These domains include knowledge, skills, beliefs about capabilities, beliefs about consequences, behavioral regulation, optimism, emotion, environmental context and resources, social/professional role and identity (hereinafter identity), intention, goal, reinforcement, social influence, and memory, attention, and decisional processes (Cane et al., 2012).

Although extended versions of TPB have incorporated additional factors such as self-efficacy, role identity, anticipated regret, moral norms, and others, these models remain limited in scope compared to the TDF, which further expands upon these constructs by integrating a broader range of behavioral influences.

**Figure 1.**
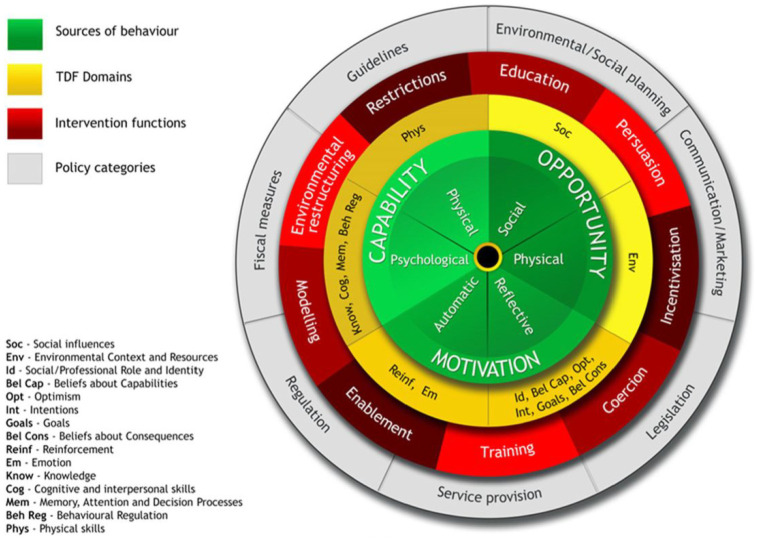
Flow chart depicting how the TDF maps onto BCW (Michie et al., 2014; Ojo et al., 2019).

After identifying the main factors that can influence an individual’s likelihood of engaging in a specific behavior, the TDF domains are aligned with intervention functions within the Behavior Change Wheel, BCW (see Figure 1; Michie et al., 2014). The BCW (Michie et al., 2014) was developed to synthesize 19 behavior change frameworks, including the TDF, through systematic literature reviews and expert consensus. The BCW offers a structured method for designing and implementing behavior change interventions, linking theoretical constructs to intervention strategies and policy categories. A distinguishing feature of the BCW is its ability to establish relationships between different elements within behavior change frameworks, ensuring a cohesive approach to intervention development.

The intervention functions are as follows: Education (providing information or increasing knowledge), Persuasion (using communication strategies to influence attitudes, beliefs or emotions), Incentivization (creating an expectation of rewards or incentives), Coercion (creating an expectation of negative outcome, punishment or threat), Training (providing instructions), Restriction (implementing rules and regulations), Environmental restructuring (modifying the physical or social environment), Modeling (providing examples or demonstrations), and Enablement (providing resources, tools or support).

The TDF has proven to be an effective tool within public health and healthcare contexts (Cane et al., 2012), offering a structured approach to understanding behavior change. Within the field of blood transfusion and donation, the TDF has been applied in cross-cultural studies to examine physician behavior regarding blood transfusions (Francis et al., 2009; Islam et al., 2012), as well as patients’ and healthcare professionals’ perceptions of transfusions (Volkmer, 2019). More recently, it has been used to investigate barriers and facilitators to domestic plasma donation in Canada (Vesnaver et al., 2023) and globally (Etherington et al., 2023). However, despite its broad application, the TDF has not yet been used to examine donor perceptions of blood donation. This presents a significant gap in the literature, as understanding how donors perceive and experience blood donation through the TDF could build upon previous research and thus, provide valuable insights for developing targeted, evidence-based interventions to improve donor recruitment and retention.

## Current study

This study aims to build upon previous research on the barriers and facilitators associated with blood donation by integrating a comprehensive theoretical framework into intervention development. Specifically, it investigates the key factors influencing blood donation behavior among young individuals in the UK, utilizing the TDF to structure the survey and identify modifiable determinants of donation behavior.

A key objective of this study is to identify modifiable factors that can be targeted to promote blood donation, highlighting specific behavioral domains that can inform future interventions mapped on BCW. Based on the findings, behavior change recommendations are proposed in the discussion.

## Method

### Recruitment

Recruitment occurred during the period of June to July 2020. Given the exploratory nature of the research, the study aimed to include at least 100 participants, a practice consistent with previous exploratory studies using the TDF (Gillman et al., 2018). Participants were recruited using a convenience sampling through multiple channels including study swap platforms, personal networks, and university recruitment documents. The inclusion criteria were age-related (targeting 18–29 years old individuals). Potential participants accessed an anonymous online survey hosted in Opinio by clicking on the provided link. No incentives were provided for participation.

This study was ethically approved by the UCL Research Ethics Committee on 11.06.2020 under the ID: 17409/001.

241 individuals responded to this survey, but 46 were excluded from the quantitative analysis (7 did not give full consent, 6 did not meet the inclusion criteria, 22 did not respond to the TDF questions, 11 responded to less than 50% of the TDF domains). This left 195 participants to be analyzed, aged between 18 and 29 (*M* = 23.33; SD = 2.57). The majority of participants were female (*N* = 147), and either a student (*N* = 105) or in full time employment (*N* = 67). Of the sample, 69 had donated blood previously whilst 126 were non-donors. Full details of the sample characteristics are presented in Table 1 and Figure 2 in the Supplemental File included a power calculation of the sample.

**Table 1.** Participants’ characteristics.

Demographics	All	Donors	Non-donors
Number	*N* = 195	*N* = 69	*N* = 126
Age	*M* = 23.33; SD = 2.57	*M* = 23.74; SD = 2.74	*M* = 23.11; SD = 2.45
Gender			
Female	147	49	98
Male	46	19	27
Other	2	1	1
Employment			
Student	105	36	69
Full time employment	67	30	37
Part time employment	16	3	13
Unemployed	7	0	7

### Design and procedure

This study employed a cross-sectional online survey. Upon clicking the provided link to access the questionnaire, participants were presented with an information sheet and were required to provide informed consent before anonymously completing the survey questions.

### Survey materials

The survey was specifically developed for this study and had not been previously validated. All questionnaire items are listed in Table 1 in the Supplemental File.

Respondents were initially asked to provide demographic information such as age, gender, current employment status, and donation history. Those who had not previously donated were subsequently asked if they would like to register to donate now, using a 5-point Likert scale. Following this, participants were provided with a registration link and were given the option to register. Finally, participants were asked whether they had indeed registered.

Next, participants were presented with statements associated with blood donation based on all 14 of the TDF domains (Table 1 in Supplemental File; Cane et al., 2012). The questionnaire was constructed based on TDF recommendations (Michie et al., 2014), previous TDF surveys, interview topic guides (Amemori et al., 2011; Gainforth et al., 2016; Smith et al., 2019), and previous blood donation questionnaires (Giles and Cairns, 1995; Lemmens et al., 2010). The survey follows a structured approach where key behavioral components - action, context, time, and target—are systematically incorporated into item phrasing, a format that has been validated for discriminant content validity in TDF implementation research (Huijg et al., 2014). Participants were asked to indicate their level of agreement with each statement on a 5-point Likert scale, ranging from 1 for strongly disagreed to 5 for strongly agreed. An exception to this was the single question addressing the Skills domain, which asked “Do you need a skill to give blood?,” where 1 represented skill needed and 3 represented skills not needed. Subsequently, participants were provided with an opportunity for free-text input to specify any skills they believed were necessary for blood donation. Supplementary questions were included for the knowledge, social influence and memory, attention and decisional processes domains. Specifically, participants were asked seven questions to test their knowledge (Figure 1 in the Supplemental File), whether they knew anyone who donated, when and where they had seen blood donation adverts.

### Analysis

The raw data was exported from Opinio, coded and analyzed using SPSS version 26.

Cronbach’s alpha was used to study the internal consistency of responses within each of the TDF domains. Descriptive statistics, such as frequencies and percentages, were used for the demographic information as well as the behavioral measures of donation experience and self-reported registration by non-donors. Descriptive statistics were also used for the additional questions for the knowledge, social influence and intention domains, in order to generate a more holistic image of participant awareness of blood donation.

To identify the key barriers and facilitators to donating blood, a mean score for each of the statements was created. Mean domain scores were also created for each TDF domain (Table 2 in text and Table 1 in Supplemental File), which were then entered into a multivariate binary logistic regression analyses to identify which domains predicted participants’ donation experience, as has been done previously in both blood donation (Holdershaw et al., 2011) and using the TDF (Isenor et al., 2018).

**Table 2.** Descriptive statistics and reliabilities of the domains.

TDF domains	Cronbach’s alpha	Mean	SD
Memory, attention, and decisional processes	0.42	3.45	0.91
Beliefs about consequences	0.74	4.17	0.62
Knowledge	0.68	3.69	0.99
Environmental context and resources	0.80	3.15	1.09
Identity	0.41	3.98	0.68
Intention	0.77	3.52	1.05
Reinforcement	0.71	3.50	0.60
Goals	0.72	2.84	0.99
Social influence	0.73	2.98	0.71
Beliefs about capabilities	0.70	3.51	0.99
Optimism	0.35	4.05	0.73
Emotion	0.81	3.32	0.82
Behavioral regulation	0.84	2.93	1.14
Skills	-	2.84	0.48

A binary logistic regression using the Forward: LR (Likelihood Ratio) method was conducted due to the large number of independent variables. To ensure model stability and avoid overfitting, the Events Per Variable (EPV) criterion was considered. With 69 donor cases and three independent variables in the final model, the EPV remained above the commonly recommended threshold of 10 events per predictor (Peduzzi et al., 1996), supporting the reliability of the logistic regression estimates.

### Developing recommendations using the BCW

Once the significant predictors were identified from the logistic regression analysis, they were mapped onto the nine intervention functions from the BCW (Figure 1; Michie et al., 2014). Choosing the intervention functions required an evidence-based judgment, taking into account affordability, practicality, effectiveness, cost-effectiveness, acceptability, side-effects/safety and equity, known as the APEASE criteria (Michie et al., 2014).

## Results

A chi-square test revealed no significant difference in gender (*X*^2^(3) = 3.35, *p* = 0.34), employment (*X*^2^(3) = 6.18, *p* = 0.10) or age (*X*^2^(11) = 9.63, *p* = 0.56) between donors and non-donors. Specifically, the participants in both groups were mostly aged between 21 and 23, female and a student.

Of the 126 non-donors, 45.4% (*N* = 57) agreed to wanting to register to become a blood donor on the day, but when presented with the registration link only 26.2% (*N* = 33) claimed to have actually registered. A chi-square test revealed no significant differences in gender (*X*^2^(2) = 0.67, *p* = 0.72) or employment (*X*^2^(3) = 2.68, *p* = 0.44) amongst those who reported not registering to become a blood donor. Specifically, for both groups participants were mostly aged between 21 and 24, female and studying.

40% of participants had seen an advert within the last month, with social media (*N* = 121) and radio (*N* = 178) being the most frequently seen. In addition, 88.1% of participants knew someone who has donated blood previously and only 34.1% said they would choose to donate this week or month.

The majority (69.7%) of non-donors indicated uncertainty in response to the knowledge questions (Figure 1 in Supplemental File). In contrast, donors exhibited significantly higher awareness of the practice of receiving a drink before donation (*X*^2^(2) = 29.34, *p* < 0.001), having their iron levels checked (*X*^2^(2) = 0.300.12, *p* < 0.001), not filling two bags of blood (*X*^2^(2) = 23.53, *p* < 0.001), not being contacted if something is found in their blood (*X*^2^(2) = 14.382, *p* = 0.001) and not undergoing a sexual health check prior to donating (*X*^2^(2) = 34.03, *p* < 0.001). There was not a significant difference between the two groups with regards to their knowledge of the duration required for blood donation (*X*^2^(2) = 10.88, *p* = 0.09), with the majority of both groups perceived the process to take between 11 and 30 minutes.

Furthermore, 12 participants responded to the question: “What skills are needed to give blood?” Specifically, three participants were not sure, four referred to being healthy and five referred to the ability to not fear needles and handle emotions.

### Reliability

Cronbach’s alpha reliability analyses were carried out for each domain with two or more questions, in order to estimate internal consistency (Table 2). The internal consistency of 9 of the 13 domains were reasonable, scoring above 0.63 (Amemori et al., 2011; Isenor et al., 2018). Behavioral regulation and social influence were the only two domains that retained all questions. For the remaining three domains, the Cronbach’s reliability analyses were particularly low, specifically optimism (*α* = 0.35), identity (*α* = 0.41) and memory (*α* = 0.42).

### Predictors of donation

Due to the low internal reliability of three TDF domains, logistic regression analysis was conducted using the remaining 11 TDF domains (Beliefs about consequences, Knowledge, Environmental context, Intention, Reinforcement, Goal, Social influence, Beliefs about capabilities, Emotion, Behavioral Regulation, Skills) along with independent questions from the low-reliability domains (Memory, attention and decisional processes, Identity and Optimism), resulting in 17 TDF independent variables. The dependent variable was donation experience and was coded as 0 = non-donors and 1 = donors. Correlation analyses were performed using Pearson’s coefficient to evaluate the relationships between the included domains and independent questions. Significant correlations were identified and presented in Table 3.

**Table 3.** Correlations between different domains and independent questions from the domain.

TDF domains	1	2	3	4	5	6	7	8	9	10	11	12	13	14	15	16	17
1. MAD (1)	1																
2. MAD (2)	0.269**	1															
3. Identity (1)	0.223**	0.234**	1														
4. Identity (2)	0.358**	0.192**	0.308**	1													
5. Optimism (1)	0.403**	0.451**	0.259**	0.256**	1												
6. Optimism (2)	0.191**	0.122	0.366**	0.242**	0.252**	1											
7. Beliefs about consequences	0.383**	0.461**	0.285**	0.380**	0.565**	0.388**	1										
8. Knowledge	0.346**	0.259**	0.290**	0.398**	0.339**	0.295**	0.546**	1									
9. Environmental context	−0.072	0.100	−0.002	0.012	0.090	0.164*	0.131	0.167*	1								
10. Intention	0.452**	0.434**	0.314**	0.385**	0.542**	0.333**	0.592**	0.465**	0.060	1							
11. Reinforcement	0.253**	0.051	0.329**	0.364**	0.257**	0.465**	0.378**	0.312**	0.071	0.271**	1						
12. Goal	0.360**	0.358**	0.314**	0.360**	0.374**	0.346**	0.556**	0.493**	0.224**	0.650**	0.275**	1					
13. Social influence	0.299**	0.178*	0.076	0.304**	0.244**	0.197**	0.398**	0.443**	0.214**	0.353**	0.323**	0.445**	1				
14. Beliefs about capabilities	0.375**	0.210**	0.142*	0.308**	0.367**	0.330**	0.432**	0.463**	0.206**	0.486**	0.169*	0.428**	0.300**	1			
15. Emotion	−0.214**	−0.296**	0.024	0.066	−0.392**	−0.093	−0.333**	−0.253**	−0.067	−0.311**	0.108	−0.220**	−0.111	−0.331**	1		
16. Behavioral regulation	0.432**	0.405**	0.315**	0.378**	0.426**	0.304**	0.573**	0.604**	0.038	0.711**	0.245**	0.708**	0.457**	0.526**	−0.326**	1	
17. Skills	−0.017	0.112	−0.072	0.009	0.042	0.061	0.095	0.008	0.097	−0.003	−0.111	0.077	−0.043	0.076	−0.103	−0.020	1

MAD (1) = If I saw frequent adverts it would encourage me to donate blood, MAD (2) = Deciding whether to donate blood is difficult), Identity (1) = I want to help others in need, Identity (2) = I feel a moral obligation to donate blood, Optimism (1) = I expect more bad things to happen than good; Optimism (2) = I am confident that donating blood will help people. MAD: memory, attention, and decisional processes.

** Correlation is significant at the 0.01 level (two-tailed).

* Correlation is significant at the 0.05 level (two-tailed).

Given the large number of independent variables, a binary logistic regression was performed using the Forward: LR (Likelihood Ratio) method.

To ensure model stability and prevent overfitting, the Events Per Variable (EPV) criterion was applied. With 69 donor cases and three independent variables in the final model, the EPV (23) exceeded the commonly recommended threshold of 10 events per predictor (Peduzzi et al., 1996), supporting the reliability of the regression estimates.

At Step 1, the mean knowledge score significantly predicted donor experience, χ^2^(1) = 55.68, *p* < 0.001. At Step 2, beliefs about capabilities were added to the model, producing a significant improvement in model fit, χ^2^(2) = 70.21, *p* < 0.001. At Step 3, the addition of emotion further improved the model, χ^2^(3) = 78.36, *p* < 0.001. The final model explained 46.1% of the variance in donor experience (Nagelkerke *R*^2^ = 0.461) and correctly classified 78.5% of cases (Table 4). No multicollinearity was detected.

**Table 4.** Predictors of donor status based on the TDF and independent questions from the domains.

Step	Predictor	*B*	SE	Wald	*p*	OR	95% CI for OR
1	Knowledge	1.629	0.218	55.684	<0.001	5.10	[3.33, 7.80]
2	Knowledge	1.424	0.237	36.110	<0.001	4.15	[2.60, 6.61]
	Beliefs about capabilities	1.124	0.295	14.523	<0.001	03.08	[1.74, 5.44]
3	Knowledge	1.397	0.244	32.768	<0.001	04.04	[2.50, 6.54]
	Beliefs about capabilities	1.013	0.317	10.210	0.001	2.75	[1.48, 5.13]
	Emotion	0.731	0.256	8.152	0.004	02.08	[1.26, 3.46]

## Discussion

The primary aim of this study was to investigate the barriers and facilitators affecting blood donation among young adults in the UK using the TDF. The results identified that Knowledge, Beliefs about capabilities and Emotion were the most significant predictors of existing donation status. Additionally, out of the non-donors, nearly half expressed a desire to register as blood donors on the day, but when given the registration link, only about a quarter reported that they actually completed the registration.

The role of knowledge in influencing donation behavior is well-documented in the literature, with multiple studies highlighting its significance (Alinon et al., 2013; Bednall and Bove, 2011; Burzynski et al., 2016; Klinkenberg et al., 2018; Polonsky et al., 2012). In our study, the Knowledge domain encompassed items that assessed participants’ awareness of the significance of blood donation and their understanding of the donation process. This domain is critical as it addresses two key aspects: the motivational aspect, which relates to understanding the importance and benefits of blood donation, and the procedural aspect, which pertains to knowing what to expect during the donation process.

Our findings suggest that donors often possess greater knowledge about the donation process, likely developed through their donation experiences. This is supported by their higher accuracy in answering procedural knowledge questions. Such experiential knowledge can reinforce positive attitudes toward donation and promote repeat donations. Conversely, qualitative data from non-donors frequently cite a lack of knowledge as a significant barrier to donation. Non-donors often reported not being aware of the benefits of donation, the safety and eligibility criteria, and the steps involved in the donation process (Enawgaw et al., 2019). This gap in knowledge can lead to uncertainty and fear, deterring potential donors from taking the first step. Knowing what happens during blood donation can demystify the process and alleviate fears or misconceptions that potential donors might have. This is particularly important for first-time donors or individuals considering donating. By being familiar with the process, individuals may feel more confident and prepared, thereby increasing their likelihood of moving from non-donor to donor status.

The second significant predictor of blood donation status identified in this study is Beliefs about capabilities, which refers to an individual’s confidence in their ability to successfully donate blood. This finding aligns with previous research using the TPB, where perceived behavioral control and self-efficacy have consistently emerged as strong correlates of donation intention (Bednall et al., 2013; Liu and Han, 2023; White et al., 2023). In the context of blood donation, higher beliefs about capabilities suggest that individuals who feel more confident in their capacity to donate are more likely to do so. Moreover, similar to knowledge, prior donation experience plays a crucial role in shaping self-efficacy—individuals who have previously donated and had positive experiences tend to exhibit higher confidence in their ability to donate again, reinforcing their likelihood of becoming repeat donors. This suggests that interventions aimed at enhancing donor confidence—particularly among first-time or hesitant donors—could play a key role in increasing long-term donor retention.

The third significant predictor of blood donation status identified in this study is Emotion, with a negative association indicating that positive emotions toward blood donation are linked to actual donation behavior, whereas negative emotions, such as anxiety and fear, serve as psychological barriers. Our results are consistent with previous research highlighting the significance of emotional factors in influencing blood donation behavior (Bednall et al., 2013), and aligns with previous research, which has consistently demonstrated that anxiety and fear are among the significant inhibitors of blood donation (Chen et al., 2022; France and France, 2018; Liu and Han, 2023). Additionally, the Emotion domain in our study encompassed not only anxiety and fear, but also positive emotional responses such as feelings of satisfaction, usefulness, and a sense of responsibility associated with blood donation. These findings align with those of France et al. (2020), who demonstrated that motivational questions—particularly those emphasizing positive outcomes or encouraging personal reflection—can enhance donation motivation. This effect is largely attributed to prompting individuals to consider the emotional rewards of giving, such as the anticipated warm glow of helping others. Our results reinforce the growing consensus that emotions play a central role in shaping donation intentions, extending beyond fear-related deterrents. More recently, Balaskas et al. (2024) also found that both positive and negative emotional states significantly influence individuals’ willingness to donate blood. Their study highlights the need for targeted communication strategies that manage stress and reduce negative emotional arousal, while amplifying positive emotional engagement. In this context, positive emotional activation emerges as a key driver in encouraging voluntary blood donation.

Regarding the self-reported registration results, there is a clear intention-behavior gap as 45.4% of non-donors agreed to wanting to register to become a blood donor on the day, but when presented with the registration link only 26.2% reported to had actually registered. This aligns with previous research indicating that intentions often do not translate into actions (White et al., 2023). One explanation is that participants were lacking time in that moment to register but went onto register at a later date. However, this discrepancy underscores the importance of interventions to bridge the gap between intention and action, reinforcing the need for targeted behavior change strategies to encourage actual donor registration and participation.

### Behavior change recommendations

Understanding the barriers to blood donation is the first step to increasing donations. Next, it is important to apply the TDF to the BCW for intervention development. By mapping the Knowledge domain onto the BCW, the primary intervention function identified is Education (Michie et al., 2014, pp. 113–115). Mapping the Beliefs about Capabilities domain onto the BCW highlights several possible intervention functions, including Education, Persuasion, Modeling, and Enablement. Lastly, mapping the Emotion domain onto the BCW identifies Persuasion, Incentivization, Coercion, Modeling, and Enablement as relevant intervention functions.

Within the Education function of the BCW, an educational initiative could offer clear, step-by-step guidance on what to expect before, during, and after donation, helping to reduce uncertainty and build donor confidence. The intervention should focus on making information engaging and accessible to younger audiences. Digital content, such as interactive infographics or animated videos could simplify the donation process and dispel myths about pain, fainting, or eligibility concerns. These could be delivered through social media platforms frequently used by young adults, such as Instagram, TikTok, and YouTube, ensuring that educational campaigns reach their target audience. Providing information about health consequences could be implemented through infomercials that present both the minor side effects for donors (e.g. a small bruise, with fainting occurring in only 1% of cases) and the life-saving impact of blood donation on recipients (France and France, 2018).

The Persuasion function, mapped onto Beliefs About Capabilities and Emotion, could involve email reminders that emphasize the simplicity and impact of donation, fostering positive emotions toward blood donation. Previous research has already confirmed the efficacy of such interventions (Germain and Godin, 2015). Additionally, storytelling and testimonials from young first-time donors who overcame their fears, could make the experience more relatable and motivating, countering common hesitations. An effective persuasion strategy to promote blood donation could involve leveraging anticipated regret, as prior research has shown that the emotional discomfort of missing the opportunity to donate significantly influences donation intentions (Bednall et al., 2013). Complementary findings by Martín-Santana et al. (2020) suggest that communication campaigns should emphasize both the positive emotions associated with donating—such as happiness, pride, and satisfaction—and the negative emotions linked to not donating, including guilt, disappointment, and self-directed anger. To further reduce resistance, campaigns should aim to alleviate negative emotions tied to the act of donation itself, such as anxiety or worry. Moreover, fostering positive attitudes toward blood donation is essential, as these attitudes play a critical role in shaping emotional responses and behavioral intentions. Building on this evidence, communication strategies could incorporate emotionally engaging narratives that prompt potential donors to reflect on how they might feel if they had the chance to donate but chose not to. In line with previous research (Balaskas et al., 2024; Conner et al., 2013; France et al., 2020; Martín-Santana et al., 2020), such campaigns should highlight both anticipated warm glow and anticipated regret. For example, testimonials from past donors expressing feelings of relief and fulfillment could be contrasted with stories from individuals who regretted not donating when they had the opportunity.

For Enablement, which is relevant to Beliefs About Capabilities and Emotion, several practical measures could be introduced to reduce barriers to donation. A pre-donation support service, such as a hotline or chatbot, integrated into messaging apps like WhatsApp, could provide real-time reassurance and answer any last-minute donor concerns. Flexible scheduling options, including extended hours and weekend appointments, could accommodate more donors and remove logistical constraints. Additionally, improving the donation environment—by making it more comfortable and welcoming—aligns with previous research on the importance of service quality in donor experiences (Mugion et al., 2021). A donation buddy system, where first-time donors are paired with experienced donors, could also serve as a social support mechanism, helping new donors feel reassured and confident.

Incentivization, a key intervention function for Emotion, could be implemented through recognition programs, such as rewarding repeat donors with certificates and badges. Gamification strategies, such as a points-based reward system, could motivate donors by offering small incentives, like free coffee or vouchers. Other exclusive donor perks, such as discounts at popular youth-focused brands or free event access, could further enhance engagement and encourage repeat donations. Such non-monetary incentives have been already supported by evidence in the context of voluntary blood donation systems (Chell et al., 2018). Social media acknowledgment, where donors receive a customized thank-you post or badge they can share on Instagram or TikTok, could reinforce positive reinforcement and help normalize donation among peer groups, also fostering social spillovers which are proven effective in previous studies (Bruhin et al., 2020).

The effectiveness of digital tools for donor engagement has already been demonstrated (Müller and Reuter-Oppermann, 2024), suggesting that an integrated donor app offering badges, rankings, and personal milestone tracking would be a practical and engaging solution for young adults. Prior research supports this approach—Sardi et al. (2019) developed a hybrid mobile application, *Blood4Life*, which integrates gamification elements with a theory of behavior change to motivate users at various stages of readiness to donate. Similarly, Sari et al. (2024) introduced *G-BlooD*, a gamified blood donation app designed using a user-centered methodology, which includes features like donation history tracking, leaderboards, and challenges. The app showed high usability and motivation scores, particularly among younger generations. These findings reinforce the potential of gamified digital platforms to foster sustained donor engagement, particularly by targeting emotional and behavioral triggers relevant to younger, tech-savvy audiences. Adding further support, Edwards and Masser (2024) examined the *Blood Harvest* campaign, where blood donation was incentivized through in-game rewards in *Diablo IV*. Analysis of social media posts showed positive reactions, community motivation, and reputational benefits, highlighting the potential of game-integrated campaigns to engage young, digitally native donors. While some limitations were noted, the study demonstrates how gaming platforms and virtual incentives can effectively promote blood donation.

Together, these findings reinforce the potential of gamified digital platforms to foster sustained donor engagement by targeting emotional and behavioral triggers that resonate with younger, tech-savvy audiences.

Although Coercion is an identified intervention function within Emotion, it is not applicable in this context, as blood donation is entirely voluntary, and any coercive measures would undermine the ethical principles of voluntary contribution.

Lastly, Modeling, which applies to Beliefs About Capabilities and Emotion, could be leveraged to increase self-efficacy by making the donation process feel more familiar and accessible. It could be particularly effective for younger audiences, who are heavily influenced by social norms, peer behavior, and digital role models. Modeling involves “providing an example for people to aspire to or imitate” (Michie et al., 2014), which in the context of blood donation is affordable, practical, acceptable, safe and cheap). Live or recorded donation demonstrations featuring real donors, student ambassadors, or relatable influencers could increase self-efficacy and foster positive feelings toward donation. NHSBT has previously used celebrities such as the England Rugby Team, radio DJs, and reality TV stars to encourage donations (for more information, see: NHSBT campaigns). However, while celebrities can be seen as credible sources of information (Goldsmith et al., 2000), they carry a large risk regarding transference of negative information from the brand (Amos et al., 2008; Till and Shimp, 1998). Furthermore, findings from a comprehensive systematic scoping review indicate that celebrities are not considered the most credible figures in a health-related context (Demeshko et al., 2022). Instead, health and medical professionals were regarded as the most trusted by the public, particularly concerning noncommunicable disease prevention. However, it is worth mentioning that in certain community settings and among specific populations, peers emerge as the most reliable spokespersons. Therefore, while modeling through celebrity endorsements can be part of a broader strategy, integrating more trusted figures such as medical professionals and peers can enhance credibility and effectiveness in promoting blood donation.

### Limitations

This research is not without its limitations. Firstly, the small sample size may not accurately reflect the broader population. Additionally, while the study focused on young adults, replicating it across diverse age groups could provide a more comprehensive understanding of the blood donation behavior in the UK. Furthermore, the study lacks representation of minority groups. Investigating young adults among minorities could reveal different barriers and facilitators, and could lead to a different intervention design. Another important consideration is the observed low reliability in certain survey domains, which may stem from the limited number of items within these domains—a recognized limitation of the TDF questionnaire (Amemori et al., 2011; Huijg et al., 2014). Given the TDF’s incorporation of various theories, its questionnaire encompasses numerous domains and their underlying constructs. Consequently, some domains may not be fully represented with all items in effort to develop a shorter version, potentially leading to the omission of some important constructs. Additionally, the lack of specification regarding the relationships between domains could contribute to overlapping, as evidenced in prior studies (Huijg et al., 2014). To address these limitations, additional exploratory research is needed. Including more items per domain and examining the patterns and associations between different domains and constructs is required. The implementation of all these steps could serve as an important foundation for future research aimed at developing a comprehensive questionnaire on blood donation based on the TDF (McGowan et al., 2020). Finally, this study is exploratory in nature, focusing primarily on investigating blood donation intentions rather than actual behaviors. The results identified associations rather than causation. As such, the findings may not fully capture the complexities of real blood donation behaviors. Further research involving the implementation of an intervention is needed to gain a deeper understanding of actual blood donation behavior in real-world settings.

## Conclusion

This study explores the key barriers and facilitators influencing blood donation among young adults in the UK using the TDF, identifying Knowledge, Beliefs about Capabilities, and Emotion as major determinants of donation behavior. A significant intention-behavior gap was observed, with nearly half of non-donors expressing a willingness to register, yet far fewer actually completing the process. To address this gap, the BCW was applied to propose affordable, practical, and cost-effective interventions. Among these, digital engagement strategies—including social media campaigns on platforms popular with young adults (e.g. Instagram, TikTok), mobile apps, chatbot support, donation tracking, and gamified reward systems—were suggested. By aligning interventions with young adults’ digital habits and social influences, blood donation initiatives can foster a more engaging, supportive, and sustainable culture of donation for future generations.

## Supplemental Material

sj-docx-1-hpq-10.1177_13591053251346387 – Supplemental material for Identifying and overcoming barriers and facilitators to blood donation in young adults using the theoretical domains frameworks Supplemental material, sj-docx-1-hpq-10.1177_13591053251346387 for Identifying and overcoming barriers and facilitators to blood donation in young adults using the theoretical domains frameworks by Velina Hristova, Freya Mills and Ivo Vlaev in Journal of Health Psychology

sj-png-2-hpq-10.1177_13591053251346387 – Supplemental material for Identifying and overcoming barriers and facilitators to blood donation in young adults using the theoretical domains frameworks Supplemental material, sj-png-2-hpq-10.1177_13591053251346387 for Identifying and overcoming barriers and facilitators to blood donation in young adults using the theoretical domains frameworks by Velina Hristova, Freya Mills and Ivo Vlaev in Journal of Health Psychology

sj-png-3-hpq-10.1177_13591053251346387 – Supplemental material for Identifying and overcoming barriers and facilitators to blood donation in young adults using the theoretical domains frameworks Supplemental material, sj-png-3-hpq-10.1177_13591053251346387 for Identifying and overcoming barriers and facilitators to blood donation in young adults using the theoretical domains frameworks by Velina Hristova, Freya Mills and Ivo Vlaev in Journal of Health Psychology
