# Supplementary material for: Identifying and overcoming barriers and facilitators to blood donation in young adults using the theoretical domains frameworks
Source: J Health Psychol. 2025 Jun 30;31(4):1692–709. doi: 10.1177/13591053251346387 (PMC12960760; doi:10.1177/13591053251346387)

**Supplementary file**

| **Table 1:**  ***Theoretical Domains Framework Survey*** | | | | | |
| --- | --- | --- | --- | --- | --- |
| Question | Statement Score | | Domain  (Reliability) | Domain Score | |
|  | Mean | SD |  | Mean | SD |
| If I saw frequent adverts it would encourage me to donate blood | 3.48 | 1.04 | Memory, attention and decisional processes  (*α =* .42) | 3.45 | .91 |
| Deciding whether to donate blood is difficult ***** | 3.42 | 1.24 |  |  |  |
| If I donated blood it would save someone’s life | 4.29 | .72 | Beliefs about Consequences  *(α =* .74) | 4.17 | .62 |
| If I donated blood, I would feel proud | 4.24 | .75 |  |  |  |
| If I donated blood, I would feel satisfied | 4.18 | .75 |  |  |  |
| The benefits of blood donation will outweigh the costs for me | 3.97 | 1.04 |  |  |  |
| I am well informed about the importance of blood | 3.94 | .97 | Knowledge  (*α =* .68) | 3.69 | .99 |
| I am familiar with what happens when you go to donate blood | 3.44 | 1.29 |  |  |  |
| Blood donation centres are in convenient places for me | 3.84 | 1.17 | Environmental context and resources  (*α =* .80) | 3.15 | 1.09 |
| Blood donation centres are open at convenient times for me | 3.65 | .97 |  |  |  |
| It is difficult for me to find time to donate blood ***** | 3.27 | 1.22 |  |  |  |
| I want to help others in need | 4.43 | .57 | Identity  (*α =* .41) | 3.98 | .68 |
| I feel a moral obligation to donate blood | 3.53 | 1.07 |  |  |  |
| How strong is your intention to give blood? | 3.21 | 1.04 | Intention  (*α =* .77) | 3.52 | 1.05 |
| Would you like to register to donate blood now? | 3.20 | 1.20 |  |  |  |
| If I donate blood, I will get recognition from my family, who are important to me | 2.82 | .91 | Reinforcement  (*α =* 71) | 3.50 | .60 |
| If I donate blood, I will get recognition from my friends, who are important to me | 2.96 | .98 |  |  |  |
| If I donate blood, I will feel like I am making a difference | 4.09 | .66 |  |  |  |
| If I donate blood, I will feel good about myself | 4.09 | .66 |  |  |  |
| Donating blood is a priority for me | 3.10 | 1.07 | Goals  (*α* = .72) | 2.84 | .99 |
| I have a clear plan of when I will donate blood | 2.57 | 1.17 |  |  |  |
| People important to me think that I should donate blood | 2.96 | .98 | Social Influence  (*α =* .73) | 2.98 | .71 |
| My friends regularly donate blood | 2.93 | 1.26 |  |  |  |
| My family regularly donate blood | 2.60 | 1.24 |  |  |  |
| If I needed help donating blood, I know someone who would help me | 3.52 | 1.12 |  |  |  |
| I talk about blood donation with people I am close to | 2.92 | 1.24 |  |  |  |
| I would donate blood if my family did | 3.08 | 1.06 |  |  |  |
| I would donate blood if my friends did | 2.90 | 1.05 |  |  |  |
| If I wanted to, I could easily donate blood | 3.38 | 1.18 | Beliefs about Capabilities  (*α* = .70), | 3.51 | .99 |
| If I wanted to, it would be easy for me to make an appointment | 3.63 | 1.06 |  |  |  |
| I expect more bad things to happen than good ***** | 3.75 | 1.15 | Optimism  (*α =* .35), | 4.05 | .73 |
| I am confident that donating blood will help people | 4.35 | .65 |  |  |  |
| The thought of donating blood makes me feel anxious | 2.95 | 1.36 | Emotion  (*α* = .81) | 3.32 | .82 |
| The thought of seeing a needle makes me feel nervous | 2.92 | 1.46 |  |  |  |
| The thought of going to a place I don’t know makes me feel nervous | 3.22 | 1.33 |  |  |  |
| I have a fear of the unknown | 2.96 | 1.23 |  |  |  |
| The thought of donating blood makes me feel uncomfortable | 3.37 | 1.30 |  |  |  |
| The thought of donating blood makes me feel useful* | 4.02 | .77 |  |  |  |
| The thought of donating blood makes me feel satisfied* | 3.82 | .84 |  |  |  |
| Signing up to donate blood is something I would do without thinking | 3.27 | 1.33 | Behavioural Regulation  (*α* = .84) | 2.93 | 1.14 |
| I have a clear plan of when I will donate blood | 2.64 | 1.25 |  |  |  |
| I have a clear plan of where I will donate blood | 2.83 | 1.31 |  |  |  |
| Do you think you need skills to donate blood? | 2.84 | .48 | Skills | 2.84 | .48 |
| Note: ***** = reverse coded |  |  |  |  |  |

**Figure 1:** ***Number of correct responses by donors and non-donors to the knowledge questions***

*Note: *** significant at p < .001
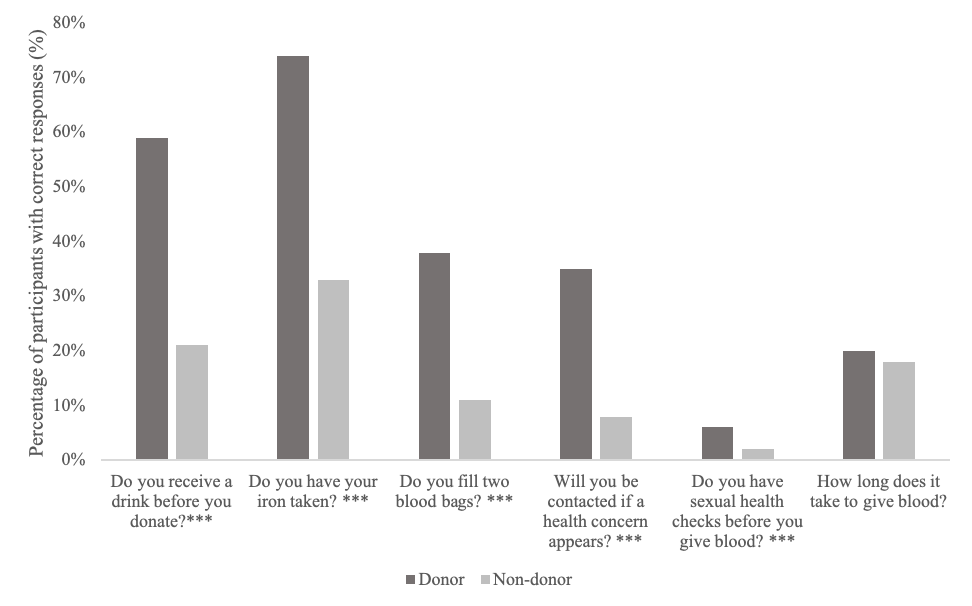
*

**Figure 2: *Results from power analysis via clincalc.com***


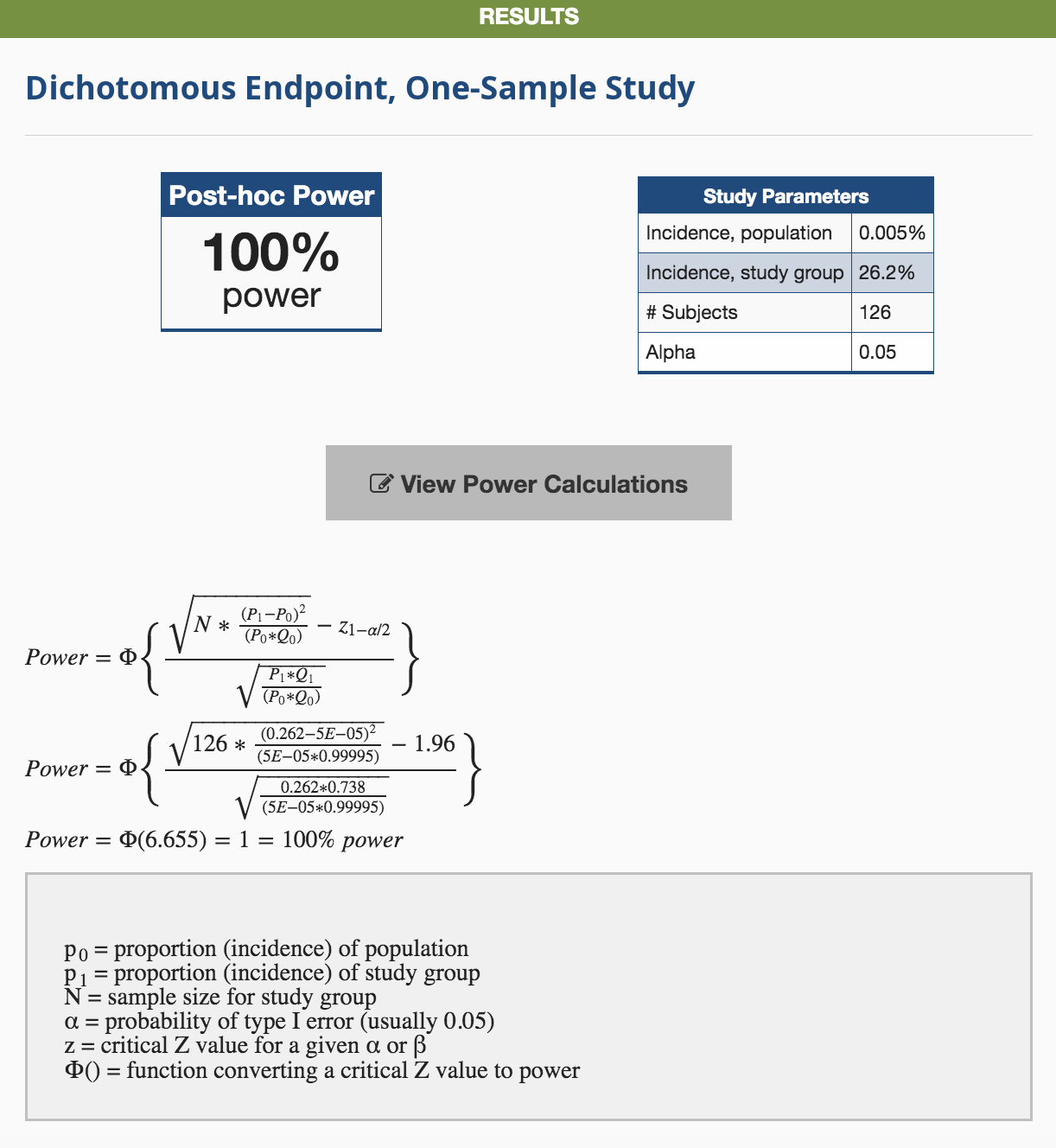

Supplement: sj-docx-1-hpq-10.1177_13591053251346387 – Supplemental material for Identifying and overcoming barriers and facilitators to blood donation in young adults using the theoretical domains frameworks [file sj-docx-1-hpq-10.1177_13591053251346387.docx]
