# Supplementary figures and images for: Identifying and overcoming barriers and facilitators to blood donation in young adults using the theoretical domains frameworks
Source: J Health Psychol. 2025 Jun 30;31(4):1692–709. doi: 10.1177/13591053251346387 (PMC12960760; doi:10.1177/13591053251346387)

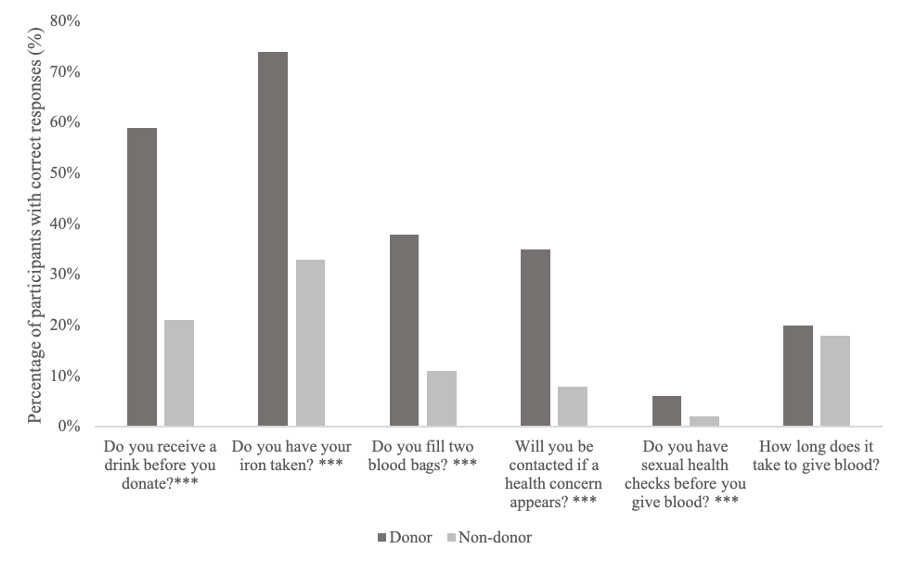

Supplement: sj-png-2-hpq-10.1177_13591053251346387 – Supplemental material for Identifying and overcoming barriers and facilitators to blood donation in young adults using the theoretical domains frameworks [file sj-png-2-hpq-10.1177_13591053251346387.png]

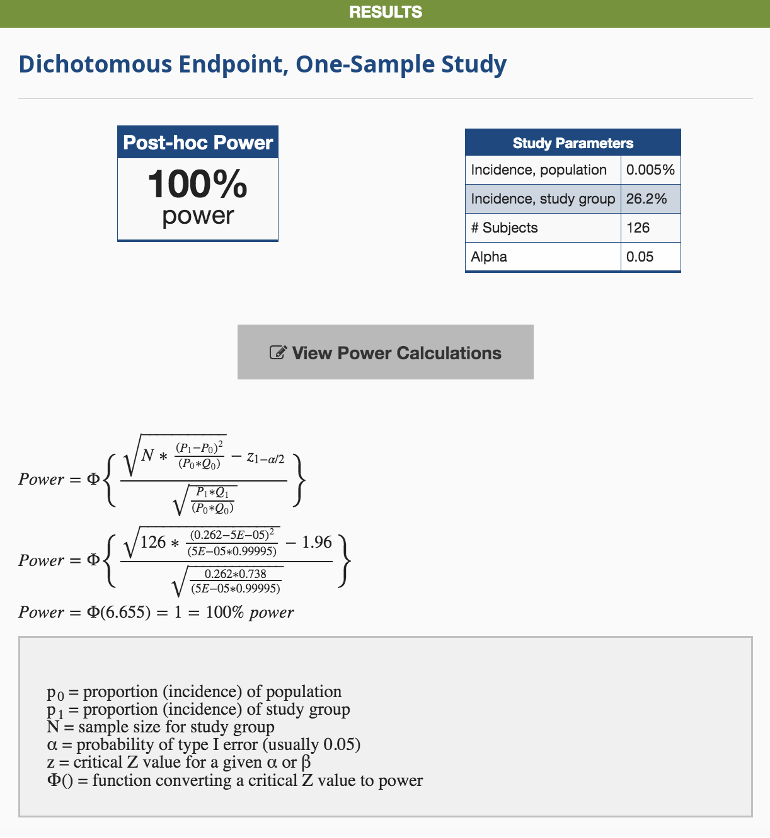

Supplement: sj-png-3-hpq-10.1177_13591053251346387 – Supplemental material for Identifying and overcoming barriers and facilitators to blood donation in young adults using the theoretical domains frameworks [file sj-png-3-hpq-10.1177_13591053251346387.png]
